# Density Fluctuations Inside an Individual Polymer Coil

**DOI:** 10.3390/polym15194018

**Published:** 2023-10-07

**Authors:** Anatoly E. Chalykh, Uliana V. Nikulova, Vladimir K. Gerasimov, Vladimir V. Matveev

**Affiliations:** Frumkin Institute of Physical Chemistry, and Electrochemistry Russian Academy of Sciences (IPCE RAS), 31, Bld.4 Leninsky Prospect, 119071 Moscow, Russia; ulianan@rambler.ru (U.V.N.);

**Keywords:** polymer coil, density fluctuation, radial distribution function

## Abstract

More than five hundred images of individual macromolecules of random styrene-butadiene copolymers and styrene-isoprene block copolymers dissolved in a polystyrene matrix were analyzed. The presence of density fluctuations inside the macromolecular coil has been established. Within the framework of the model of harmonic oscillations, the radial distribution of such density fluctuations is estimated.

## 1. Introduction

The capabilities of electron microscopy have expanded so much that when combined with various methodological approaches to the preparation of objects, it has become possible to solve issues related to detailing the structural features of individual elements included in the object [1,2]. Currently, electron microscopic equipment is being rapidly improved, and special analytical electron microscopes are being created to determine the composition of micro-objects using electronic and x-ray spectra. At the same time, the general development trend is to increase the brightness of the gun, improve the vacuum, reduce the size of the probe, and increase its stability and the use of cryotechniques. All this meets the requirements for further improving the analytical parameters of X-ray spectral attachments in electron microscopy, which opens up great prospects in the development of microanalysis of micro-objects. The study of the conformational state of macromolecular segments of polymer coils of individual macromolecules is of particular interest.

A large number of experimental and theoretical works, summarized in [3,4,5,6,7,8,9], are devoted to the study of the conformational state of polymer coils. In most cases, these studies analyzed the dependence of the radius of gyration of a macromolecule on the thermodynamic quality of the solvent, the degree of polymerization of the macromolecule, and the structure and composition of the macromolecular chain. Based on the results of these studies, a fundamental conclusion was made that the radius of gyration of a macromolecule is proportional to the square root of the molecular weight (degree of polymerization), and that “coil fluctuations have the size of a coil” [4,5], which was confirmed in subsequent experimental and methodological studies.

Processing the data of an X-ray diffraction analysis of the small angles of amorphous polymers made it possible to obtain and theoretically describe the radial distribution function of the density of segments depending on the number of segments and the radius of gyration of the macromolecule, and hence, indirectly, on the thermodynamic quality of the solvent [5]. A theoretical description of the density distribution inside a macromolecular coil has been proposed, representing it as a spherically symmetric object [10].

(1) $$\rho \left(r\right)=n{\left[\frac{3}{2\pi {\left(2R\right)}^{2}}\right]}^{\frac{3}{2}}\exp\left[-\frac{3r^{2}}{2R^{2`}}\right]$$

where n is the number of segments of the macromolecule, R is the radius of gyration of the macromolecule, and r is the current radius.

The found segment density distribution functions are in good agreement with experiment. Below, we will use this expression to describe the radial distribution function of the density of segments, denoting it as the equilibrium $\rho_{eq}\left(r\right)$.

However, X-ray studies provide results averaged over an ensemble of macromolecules. In this case, the radial density distribution function is well described using Equation (1). In order to analyze each individual macromolecule without averaging over the ensemble and to identify intra-ball density fluctuations, transmission electron microscopy should be used.

In previous works [11,12,13], we described in detail the methods for preparing samples for electron microscopy. It was also proved that the observed objects are individual macromolecular coils, their ensemble (average) characteristics were obtained, the molecular masses and radii of gyration of specific individual macromolecules were determined, and the radial density distribution functions of the segments of each macromolecule were calculated.

The purpose of this work is to study the behavior of the radial density distribution function of segments of individual macromolecules using random styrene-butadiene copolymers and styrene-isoprene block copolymers as an example and, also, to create a technique for processing images of individual macromolecules with an analysis of the relationship between their size and other structural characteristics of the coil.

## 2. Materials and Methods

The objects of study were macromolecules of random butadiene-styrene copolymers SBR-96 (Voronezhsintezkauchuk, Voronezh, Russia, M_w_ = 100 kDa, molecular weight distribution (MWD) = 1.7, and 173 macromolecules) and SBR-45 (Voronezhsintezkauchuk, Russia, M_w_ = 100 kDa, MWD = 1.8, and 157 macromolecules), as well as macromolecules of styrene-isoprene block copolymers SIS-4114 (Corium LTD, Menlo Park, CA, USA, M_η_ = 100 kDa, 15% styrene units, and 110 macromolecules) and SIS-4215 (Corium LTD, USA, M_η_ = 100 kDa, 30% styrene units, and 148 macromolecules). In total, there are 584 individual macromolecules. Polystyrene (PS) was used as a matrix in which macromolecules of copolymers were dissolved (Aldrich, Wyoming, IL, USA, M_w_ = 15 kDa and 350 kDa, MWD = 1.004).

**Figure 1:**
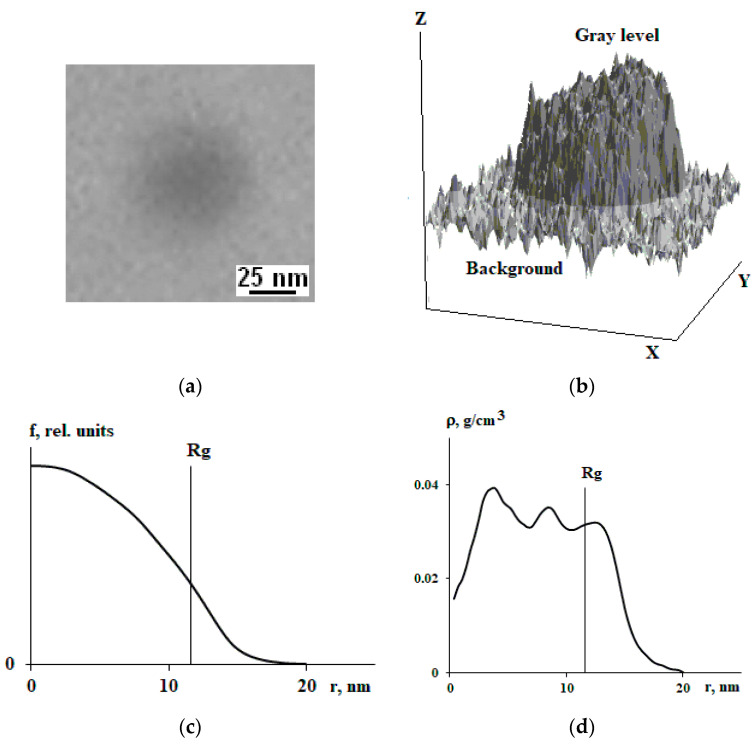
An electron microphotograph of an individual SBR-45 macromolecule in a polystyrene matrix (**a**) and the distribution of gray levels corresponding to it over the image area of the macromolecule (**b**), the blackening radial function (**c**), and the segment density radial distribution function (**d**).

PS was mixed with each of the copolymers through a solvent (toluene). The concentration of copolymers varied from 0.01 to 0.05% based on PS, which makes it possible to obtain individual macromolecules distributed in the PS matrix in the final objects. The studies were carried out on films that were prepared by pouring onto the glass surface. The measurements were carried out on films 100 µm thick, which were preliminarily annealed at a temperature slightly above the glass transition temperature of PS. Samples of mixtures were contrasted using OsO_4_ at double bonds at a temperature of 160 °C for 24 h. Preliminary studies have shown that the distribution of osmium over the cross section of the sample is uniform and does not affect the size and conformational state of macromolecules. The samples were viewed using a transmission electron microscope EM-301 (Philips, Amsterdam, The Netherlands) at a magnification of ×80,000. In this way, 584 images of individual macromolecules were studied. The digital image was recorded with an Olympus camera (Olympus Corporation, Tokyo, Japan) with an additional magnification of ×10 (Figure 1a). Converting the image into digital form allows you to obtain a matrix of gray levels from 0 to 255 (Figure 1b). By subtracting the background, it can determine the center of mass and by summation along the X and Y axes and mirror reflection relative to the center of mass to obtain a radial function of the degree of blackening f(r) (Figure 1c), uniquely related to the concentration of double bonds in copolymers contrasted with osmium. Note that in this study we can only observe isoprene blocks of styrene-isoprene block copolymer. Therefore, the molecular weight and radius of gyration values apply only to the isoprene block of the block copolymers. Using Equation (2) [14] by means of numerical differentiation f(r), we obtained the radial segment density distribution function ρ(r) (Figure 1d). If on a small interval of a curve (smooth curve) f(r) can be described by a polynomial, then the tangent at a point is equal to the angle of inclination of the secant line through two points equidistant from it.

(2) $$\rho \left(r\right)=-\frac{\frac{df\left(r\right)}{dr}}{2\pi r}$$

## 3. Results and Discussion

**Figure 2:**
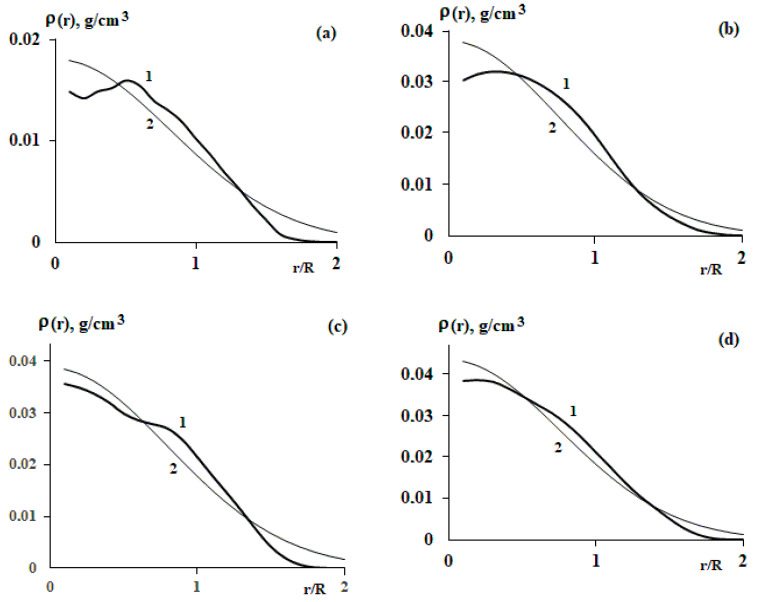
Average radial distribution functions of segment density (lines 1) for SBR-96 (**a**), SBR-45 (**b**), SIS-4114 (**c**), and SIS-4215 (**d**). Lines 2 correspond to calculations within the framework of Equation (1). The current radii are normalized to the radius of gyration.

As a result of processing all microphotographs, averaged radial distribution functions were obtained inside the macromolecular coil, where the current radius is normalized to the radius of gyration (Figure 2). It can be seen that the distribution functions are satisfactorily described using Equation (1). Therefore, when analyzing density fluctuations, (1) can be used, denoted as $\rho_{eq}\left(r\right)$ and under the assumption that there are no density fluctuations in this equation.

**Figure 3:**
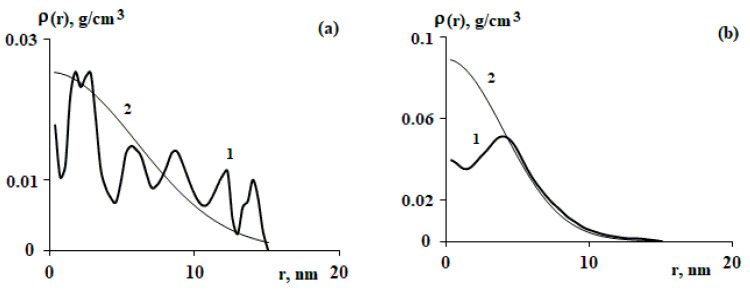

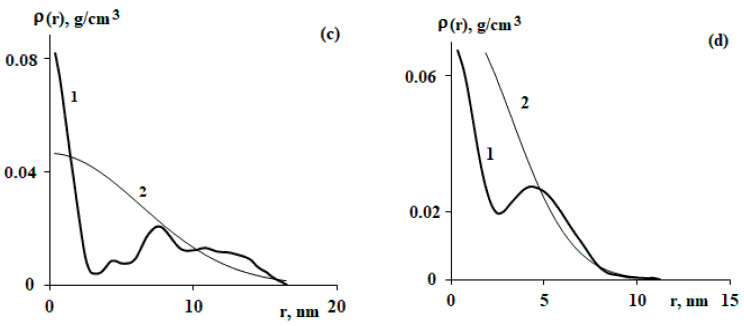
Radial distribution functions of segments for macromolecules SBR-96 (M=106.4 kDa) (**a**), SBR-45 (M = 57.6 kDa) (**b**), SIS-4114 (M = 100.9 kDa) (**c**), and SIS-4215 (M = 24.3 kDa) (**d**); 1—obtained by processing the image of a macromolecule; 2—$\rho_{eq}\left(r\right)$ according to Equation (1) with the constants (N, $R_{g}$) corresponding to each individual macromolecule. The indicated molecular weight (M) corresponds to the specific macromolecule chosen.

The radial distribution function of segments of a particular polymer chain, as a rule, is characterized by significant density deviations from $\rho_{eq}\left(r\right)$. As examples, Figure 3 shows several radial segment distribution functions and compares these values as described using Equation (1).

The analyzed images of individual macromolecules of copolymers refer to the moment when the glass transition of the polystyrene matrix occurred. Such a different behavior of the radial distribution function of the density of individual macromolecules, shown in Figure 3, is due—in our opinion—to fluctuations of a particular macromolecule at a particular point in time (the moment of fixing the copolymer structure). The commonality lies in the fact that almost all the studied macromolecular coils demonstrate fluctuation oscillations relative to $\rho_{eq}\left(r\right)$. We defined the deviations of the segment density distribution relative to the equilibrium state as density fluctuations.

It is known from [4,5] that fluctuations in the density of a polymer coil can correspond to the size of the coil. This is expressed in the size distribution of coils of the same molecular weight given, for example, in [15]. We consider density fluctuations of a much smaller size—inside a macromolecular coil. Figure 3 shows that density fluctuations are characterized by both radial size and deviation.

The expression for the radial density distribution function of segments can be represented as the sum of the equilibrium $\rho_{eq}\left(r\right)$ and fluctuation $\rho_{fl}\left(r\right)$ parts:

(3) $$\rho \left(r\right)=\rho_{eq}\left(r\right)+\rho_{fl}\left(r\right)$$

Hence, the fluctuation part of the radial density distribution function can be represented in a form more convenient for analysis:

(4) $$\rho_{fl}^{/}=\frac{\rho \left(r\right)}{\rho_{eq}\left(r\right)}-1$$

Respectively,

(5) $$\rho_{fl}^{/}=\frac{\rho \left(r\right)-\rho_{eq}\left(r\right)}{\rho_{eq}\left(r\right)}=\frac{\rho_{fl}\left(r\right)}{\rho_{eq}\left(r\right)}$$

**Figure 4:**
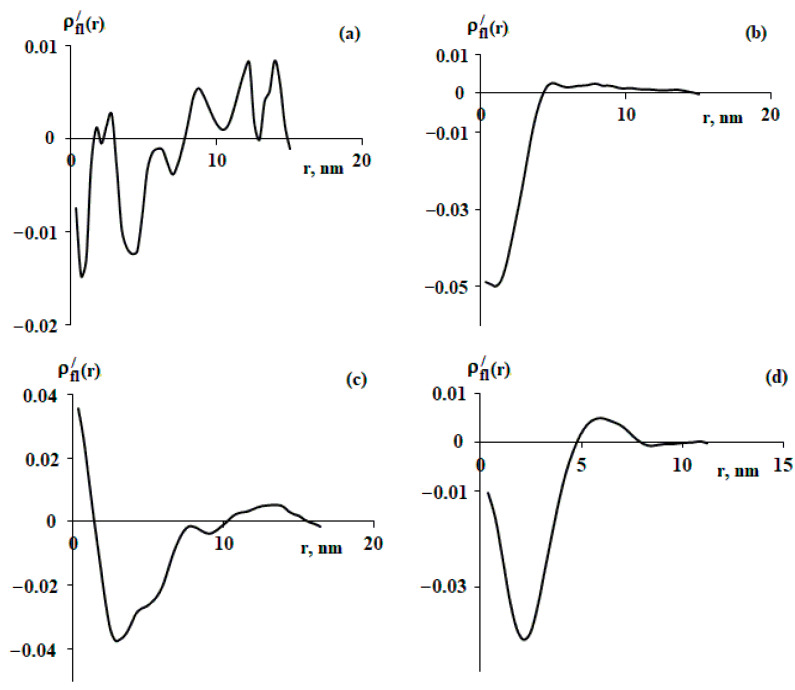
Fluctuation part of the radial density distribution functions for macromolecules shown in Figure 3. Explanations in the text.

The fluctuation parts of the radial segment density distribution functions shown in Figure 3 are presented in Figure 4. It should be noted that for a particular macromolecule, (Figure 4a) quite a lot of fluctuations of the density distribution function were recorded, which can be seen from the large number of beats. At the same time, Figure 4b, which corresponds to another macromolecule, has a small number of fluctuations.

From the analysis of the curves shown in Figure 3, it can be seen that as the distance from the center of mass increases, the fluctuation amplitude decreases. It can be assumed that it decreases in proportion to $\rho_{eq}\left(r\right)$.

An analysis of the entire set of curves $\frac{\rho_{fl}\left(r\right)}{\rho_{eq}\left(r\right)}$ allowed us to assume that in the zeroth approximation they can be described with the simplest harmonic function, which well describes the general trend of transformation of density fluctuations in dynamics:

(6) $$\frac{\rho_{fl}\left(r\right)}{\rho_{eq}\left(r\right)}=A\cos\left(\omega r+\alpha \right)$$

where A is the oscillation amplitude, ω and α are the frequency and phase shift, and r is the current radius.

In this case, the radial density distribution function of the macromolecule will have the following form:

(7) $$\rho \left(r\right)=\rho_{eq}\left(r\right)+\rho_{eq}\left(r\right)А\cos\left(\omega r+\alpha \right)$$

It should be noted that the proposed model (7) describes the radial arrangement of fluctuations, which is enclosed in a spherical layer with a thickness of 2π/ω. Therefore, we consistently record one measurement of each fluctuation.

**Figure 5:**
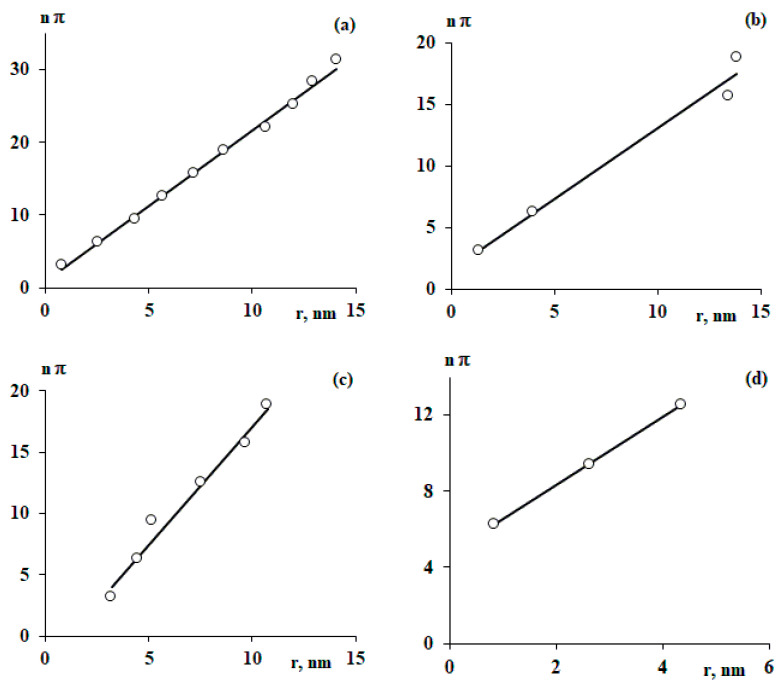
The position of the extrema of the functions shown in Figure 4. The linear dependence is described using the equation ωr+α. Explanations in the text.

It is known that the distance between the extrema of the harmonic function $\frac{\rho_{fl}\left(r\right)}{\rho_{eq}\left(r\right)}=min,max$ is proportional to π. This makes it possible to determine the parameters ω and α of the oscillatory model. As an example, Figure 5 shows the dependences of the position of the extrema of the function $\frac{\rho_{fl}\left(r\right)}{\rho_{eq}\left(r\right)}$ on the center of mass of the macromolecule.

In the case when the dependence $\frac{\rho_{fl}\left(r\right)}{\rho_{eq}\left(r\right)}$ has inflection points between extrema (as an example, Figure 5b), a period was added between them, i.e., 2π to the best description of the dependence using a linear model.

**Figure 6:**
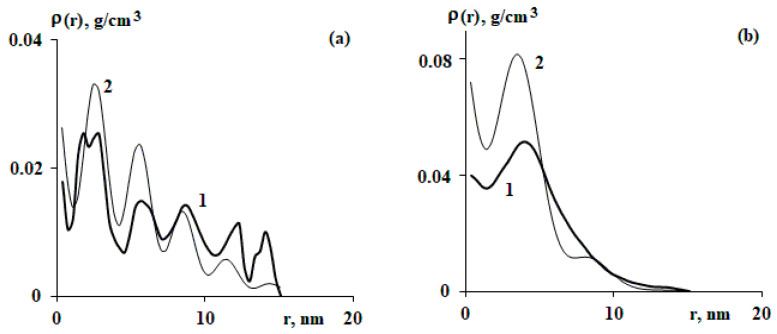

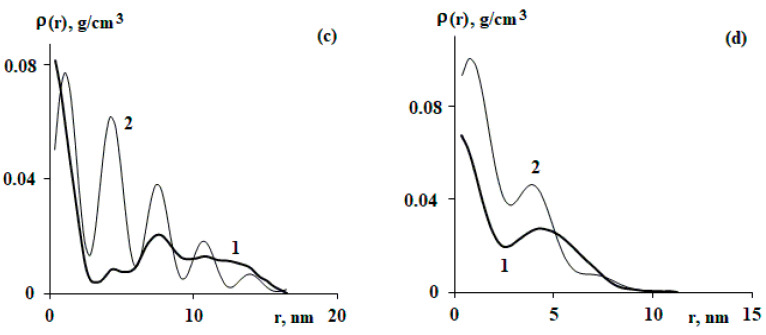
Radial segment distribution functions for copolymers whose segment density radial distribution functions are shown in Figure 3 (1); 2—description by the model of harmonic oscillations (7).

Knowing the parameters ω and α of dependence (6) allows us to determine the value of A. When averaging the amplitudes, we took the weight function equal to the reciprocal distance of the extremum from the center of mass. As an example, Figure 6 shows the results of such a description for the macromolecules shown above in Figure 3, Figure 4 and Figure 5.

It can be seen that for the zero approximation of the description of the fluctuation part of the radial distribution function of the density of segments, the agreement between the one determined from the analysis of the electron microscopic image of an individual macromolecule and the calculations using Equation (7) is satisfactory.

For the parameters of the fluctuation behavior of macromolecules A, ω, and α obtained by processing the entire array of data, it seemed interesting to reveal their possible relationship with the ensemble characteristics of the systems. The Flory–Huggins parameter χ [12,13], which reflects the interaction between the copolymer (isoprene block of SIS block copolymers) and the polystyrene matrix, and the number of segments of macromolecules (N), which reflects only the properties of the macromolecule, were chosen as such. The choice of these parameters is due to the fact that almost all other properties of polymers theoretically or empirically depend on these quantities or their combinations. The Flory–Huggins parameters for each macromolecule were previously calculated from the deviation of the radius of gyration from its equilibrium value in the θ state [12,13].

**Amplitude**

**Figure 7:**
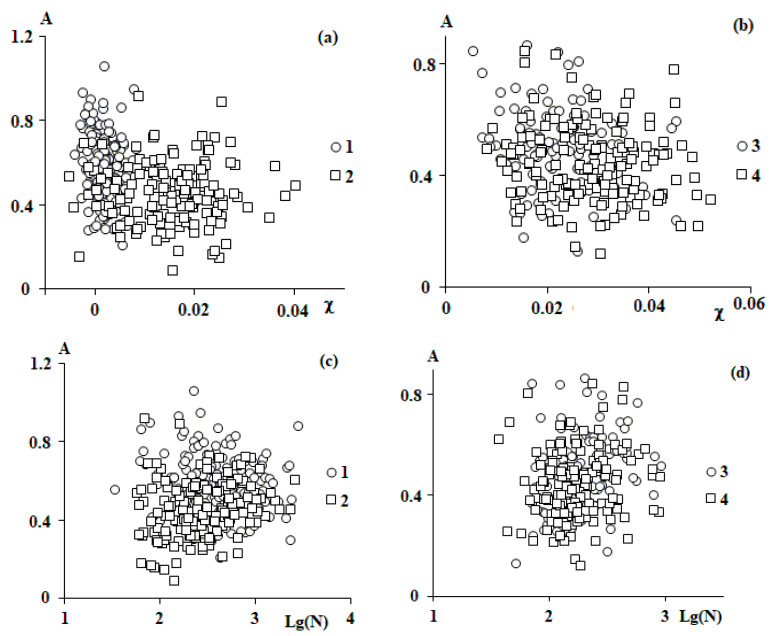
Correlation between Flory–Huggins parameter χ (**a**,**b**), number of segments N (**c**,**d**) and amplitude А for all studied individual macromolecules; 1—SBR-96, 2—SBR-45, 3—SIS-4114, and 4—SIS-4215.

Figure 7 shows the results of comparing χ and N with the amplitude of harmonic oscillations for all studied images of 584 individual macromolecules. It can be seen that the amplitude does not depend on the chain length. It is not possible to analyze the dependence of the amplitude on the length of the kinetic segment due to the fact that the kinetic segments of polystyrene, polybutadiene, and polyisoprene are close (about 2 nm [16]), and the scatter of the calculated values is significant. The dependence of the amplitude on the Flory–Huggins parameter for the polyisoprene–polystyrene system was not revealed. For styrene-butadiene copolymers, there are some reasons to assume such a dependence (Figure 7a); however, in this case, the scatter of the calculated values is significant, so it is impossible to draw an unambiguous conclusion. In addition, the copolymers of butadiene and styrene and isoprene are similar both in terms of the Flory–Huggins parameter of copolymers with polystyrene and in terms of the size of the Kuhn length.

**Figure 8:**
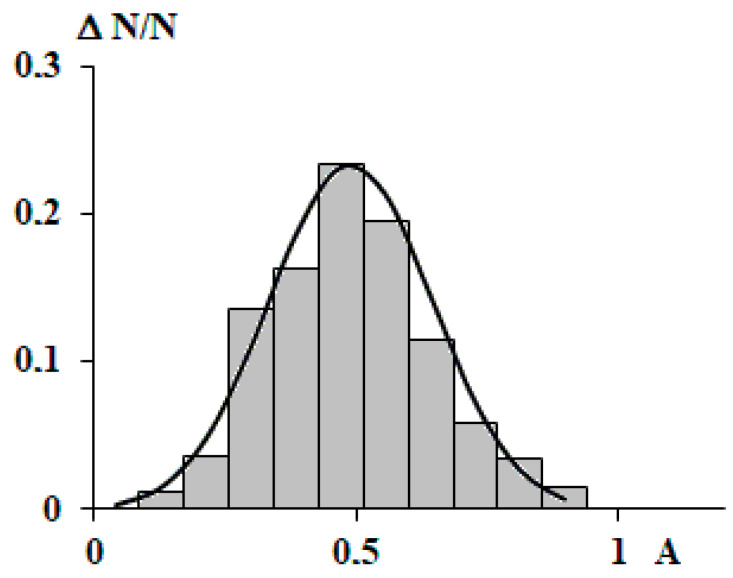
Histogram of the distribution of the amplitude of harmonic oscillations describing density fluctuations of all studied macromolecules. The continuous curve is a function of the normal logarithmic distribution.

Based on the results of the analysis of the data presented in Figure 7, it can be reasonably assumed that the amplitude is independent of the above arguments. In this case, its mean value and distribution around the mean should be estimated. Figure 8 shows a histogram of the distribution of amplitudes and its description with a normal distribution. It can be seen that the description is quite satisfactory.

Taking into account the fact that, without much loss of accuracy, the amplitude can be taken as a constant with the value 1/2, Equation (7) takes the following form:

(8) $$\rho \left(r\right)=\rho_{eq}\left(r\right)+\frac{\rho_{eq}\left(r\right)}{2}\cos\left(\omega r+\alpha \right)$$

The density of a macromolecular coil at the center of mass of a macromolecule, described using Equation (1), has the following form:

(9) $$\rho_{\mathrm{eq}}\left(0\right)=N{\left[\frac{3}{2\pi {\left(2R\right)}^{2}}\right]}^{\frac{3}{2}}.$$

Accordingly, the expression $\frac{\rho_{eq}\left(r\right)}{2}$ from Equation (8) takes the following form:

(10) $$\frac{N}{2}{\left[\frac{3}{2\pi {\left(2R\right)}^{2}}\right]}^{\frac{3}{2}}.$$

That is, the maximum size of the fluctuation of the radial density of a macromolecular coil is half its density at the center of mass.

**Frequency**

**Figure 9:**
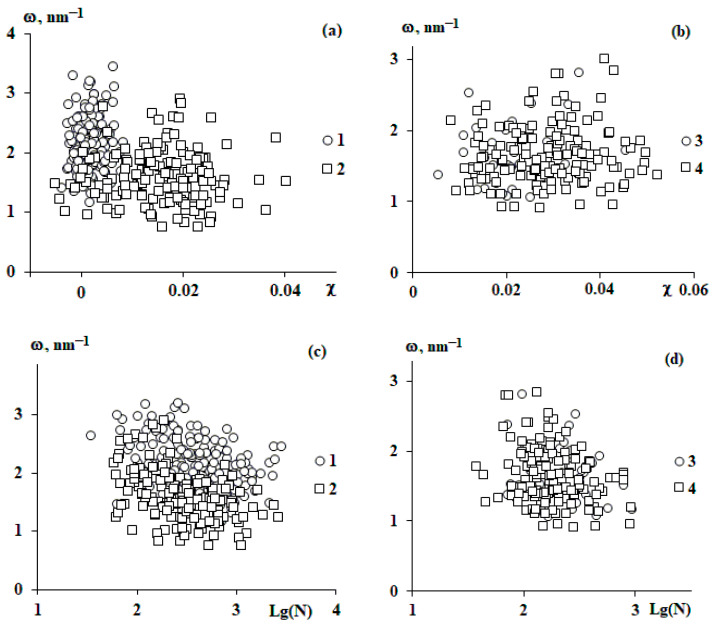
Correlation between the Flory–Huggins parameter χ (**a**,**b**), the number of segments N (**c**,**d**), and the harmonic frequency ω for all studied individual macromolecules; 1—SBR-96, 2—SBR-45, 3—SIS-4114, and 4—SIS-4215.

Figure 9 shows the results of comparing χ and N with the frequency of harmonic oscillations for all studied systems. It can be seen that there are no distinct correlations with either χ or N. This is probably due to the same reasons as in the description of the amplitude of oscillations describing density fluctuations.

**Figure 10:**
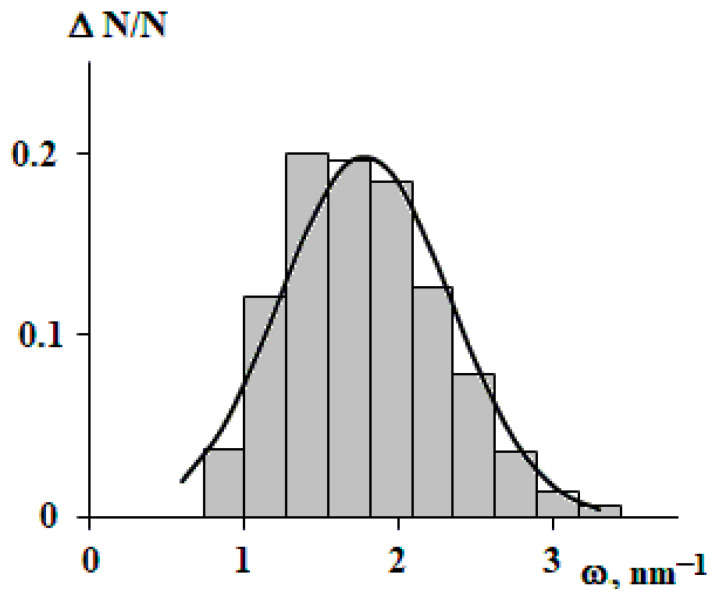
Histogram of the distribution of the frequency of harmonic oscillations describing density fluctuations for all studied macromolecules. Continuous curve is a normal distribution function.

The average frequency is 1.8 nm^−1^. The frequency distribution histogram is shown in Figure 10. Density fluctuations in the physical chemistry of polymers are commonly understood as an increase in density relative to the average. From this point of view, the period of harmonic oscillations can be considered as the size of a double density fluctuation (increase in density in the first half of the period and rarefaction in the second). The period T = 2 * π/ω is 3.5 nm. It should be noted that the size of density fluctuations is comparable with the Kuhn segments of the studied polymers and the size of the reptation loop [17]. This fact requires separate consideration and reflection.

**Phase shift**

**Figure 11:**
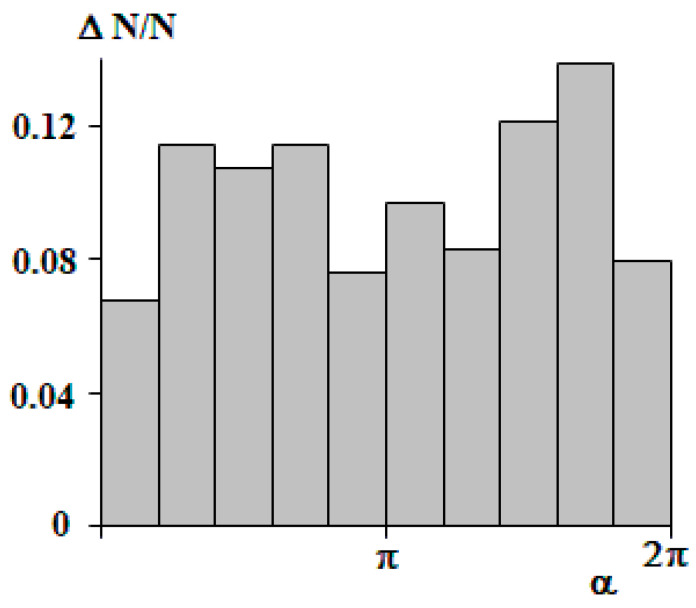
Histogram of the phase shift distribution from the center of mass.

An analysis of the possible correlation dependencies of the phase shift on the Flory–Huggins parameter or the length of the macromolecule showed their expected complete independence. Figure 11 shows a histogram of the phase shift distribution over a full cycle distance. It can be seen that the values of the phase shift are uniformly distributed over the interval [0, 2π]. This means that the first fluctuation occurs at an arbitrary distance from the center of the mass.

## 4. Conclusions

In this work, by analyzing images of 584 individual macromolecules of random copolymers of styrene and butadiene, as well as block copolymers of styrene and isoprene dissolved in a polystyrene matrix, the presence of density fluctuations inside a macromolecular coil was reliably experimentally recorded. An attempt is made to describe the radial distribution of density fluctuations using a model of harmonic oscillations. An assumption is made about what the constants of the model of harmonic oscillations applied to the description of fluctuations in the radial density of segments are related to. It is shown that the amplitude does not depend on the chain length, and its dependence on the Flory–Huggins parameter was not revealed for SIS but may take place for SBR. The frequency with the same parameters has no distinct correlations, and the phase shift behaves completely independently.

The presented analysis is a detailed procedure for processing microphotographs of individual macromolecules of copolymers, and the results obtained make it possible to evaluate the thermal fluctuation state of coils of macromolecules.

Thus, the work summarizes the results of studying the conformational structure of individual macromolecular coils using transmission electron microscopy. It can be seen that this approach makes it possible to differentiate supramolecular structures, localize their place in the structure of an object (coil), and clarify the functional role of individual structural details that make up macromolecular chains. The information obtained can be useful in studying diffusion processes, permeability, free volume problems, etc.

## Data Availability

The data presented in this study are available on request from the corresponding author.
